# USB-powered vs. self-heating warm compresses for meibomian gland dysfunction: A prospective randomized crossover clinical trial

**DOI:** 10.1371/journal.pone.0360762

**Published:** 2026-10-13

**Authors:** Ali M. Al Saqr, Moath Faisal Alharbi, Meshary Alrumizan, Ali Abusharha

**Affiliations:** Optometry Department, College of Applied Medical Sciences, King Saud University, Riyadh, Saudi Arabia; University of Hyderabad, INDIA

## Abstract

**Background:**

Dry eye syndrome (DES) is a prevalent multifactorial condition characterized by tear film instability and meibomian gland dysfunction (MGD). Warm compress therapy remains a cornerstone for improving lipid layer function. However, very limited comparative data exist on newer USB-powered devices vs. self-heating masks in Middle Eastern populations.

**Methods:**

This randomized crossover clinical trial compared USB-powered and self-heating masks in 15 participants aged 18–40 years with meibomian gland dysfunction (MGD) associated eye (tear break-up time ≤10 s). Each intervention was scheduled for five consecutive days, separated by a two-week washout. The prespecified primary outcome was non-invasive TBUT measured with the OCULUS Keratograph 4M; tear meniscus height (TMH) and symptom scores were secondary outcomes. A two-way ANOVA and Post-hoc Bonferroni tests adjusted for multiple comparisons were used for analysis. The trial was registered at ClinicalTrials.gov (NCT07618169).

**Results:**

All 15 participants contributed data to both intervention phases. Across observed visits, the USB-powered device was associated with higher TBUT (likelihood-ratio χ^2^(1) = 7.43, p = 0.006) and TMH (χ^2^(1) = 4.49, p = 0.034). Day 5 descriptive means with USB-Powered compress and self-heating mask were 9.53 ± 2.18 s versus 7.21 ± 2.90 s for TBUT, and 0.200 ± 0.020 mm versus 0.188 ± 0.016 mm for TMH, respectively. No significant between-device difference was observed in SPEED scores, although mean scores showed a mild improvement, and no adverse events were reported.

**Conclusions:**

USB-powered warm compresses provided superior outcomes in enhancing tear film stability and volume compared to self-heating masks. Consistent temperature control and reusability make them a promising at-home therapy for patients with MGD. USB-powered devices may be preferred for patients requiring sustained thermal therapy, particularly in arid climates with variable indoor cooling. Further large-scale trials are warranted to confirm these findings.

**Clinical Trial Registration:**

This trial was registered at ClinicalTrials.gov under registration number: NCT07618169.

## 1 Introduction

Dry eye syndrome (DES) is a multifactorial disease of the ocular surface characterized by loss of tear film homeostasis and accompanied by symptoms such as ocular discomfort, burning, and fluctuating vision [1]. Globally, DES affects an estimated 5% to 50% of adults, depending on diagnostic criteria and geography [2,3]. The burden is especially high in the Middle East, where hot, dry climates, high screen exposure, dust, and systemic diseases such as diabetes contribute to elevated prevalence [4–6]. Recent population-based studies in Saudi Arabia and in the middle east have reported a prevalence of clinically significant DES ranging from 32% to 55% [7–9].

In addition to its clinical burden, DES imposes a significant economic impact worldwide. A systematic review estimated that the global annual cost of managing DES including direct medical care and productivity losses exceeds USD 55 billion [10]. In the Middle East, untreated DES increases healthcare expenditure through frequent clinic visits, overuse of lubricants, and productivity losses [11], although no current studies have formally investigated the DES economic burden.

A major contributor to evaporative DES is meibomian gland dysfunction (MGD), which accounts for up to 70% of DES cases globally [12]. The MGD is characterized by ductal obstruction or qualitative and quantitative changes in meibum secretion, destabilizing the lipid layer and accelerating tear evaporation [13]. If left untreated, MGD can lead to chronic ocular surface inflammation, corneal epithelial damage, and reduced quality of life and mental health [14–16].

Warm compress therapy remains a cornerstone in the management of MGD, as it melts inspissated meibum and restores gland function [17,18]. Conventional warm compresses, such as hot towels, often fail to maintain temperatures above the therapeutic threshold of 40 °C for the duration needed to liquefy thickened meibum [19]. Recent evidence shows that although self-heating steam eye masks are convenient, they may not reliably sustain eyelid temperatures ≥40 °C throughout their use [20]. Wang et al. (2015) demonstrated that while self-heating masks (EyeGiene® Insta-Warmth Mask) can initially reach target temperatures, they lose heat rapidly, especially in variable room conditions, which may reduce clinical efficacy [21]. Despite regional DES prevalence exceeding 50% [7,8], no crossover trials have compared USB-powered compresses and self-heating masks in Middle Eastern populations, where environmental factors (e.g., air conditioning, dust) may influence device efficacy which are known to exacerbate evaporative tear loss and may modulate treatment response [1,2,7,8].

To overcome these limitations, newer modalities such as disposable self-heating mask and USB-powered electrical warm compresses have been suggested. Some of these devices offer adjustable and sustained heat and have demonstrated more consistent temperature maintenance [19]. Lee (2024) systematically reviewed eyelid warming therapies and concluded that self-heating masks showed similar short-term benefits, with variability in heat-up time and duration, although it was better than hot towels [19]. Comparative studies support this finding. Arita et al. (2015) and Olafsson et al. (2021) compared a steam-generating mask to a heated eye pad and found that the pad maintained higher eyelid temperatures and improved tear film break-up time more effectively [17,22]. Similarly, Olson and Korb (2003) observed that moist warm compresses significantly improved meibomian gland expressibility and increased lipid layer thickness compared to room-temperature controls [23]. Previous evidence supports both Alghamdi et al. (2017) and Chuckpaiwong et al. (2022) findings who suggested that patient compliance and consistent use of eyelid hygiene or warming therapies were strongly associated with symptom relief in meibomian gland dysfunction, underscoring the importance of practicality, comfort, and sustained heat delivery [24,25].

Given the high prevalence and economic burden of DES in Saudi Arabia and the broader Middle East, there is an urgent need for robust comparative data to guide home-based MGD therapies. To date, no randomized trials have directly compared USB-powered warm compresses with self-heating masks in a crossover design. This study addresses this evidence gap by evaluating the relative efficacy of these two modalities in improving tear film stability, tear volume, and patient-reported symptoms among individuals with DES and MGD. We hypothesize that USB-powered compresses, with their adjustable, sustained heat delivery, will demonstrate superior outcomes, supporting evidence-based recommendations for effective dry eye management. This study would not only evaluate clinical outcomes but also patient compliance under typical Middle Eastern living conditions, addressing a critical evidence gap.

## 2 Materials and methods

### 2.1 Ethical considerations and trial registration

This study protocol was approved by the King Saud University Institutional Review Board (IRB No. E-25–9589) before participant recruitment began. All procedures adhered to the Declaration of Helsinki and its later amendments, and written informed consent was obtained from all participants. Confidentiality and anonymity were maintained throughout the study. This clinical trial was registered at ClinicalTrials.gov under registration number: NCT07618169. The trial was registered after recruitment began because the investigators initially classified it as a prospective comparative interventional study of two commercially available devices used in clinical practice. The authors confirm that all ongoing and related trials for this intervention are registered.

### 2.2 Study design

This prospective, single-centre, randomized crossover clinical trial was conducted at the Department of Optometry, King Saud University, Riyadh, Saudi Arabia. Participant recruitment was conducted from July 15, 2025, to October 30, 2025. Follow-up and completion of both intervention phases were conducted between July 2025 and December 2025. Participants were randomized to receive two eyelid warming interventions in two sequential phases, separated by a two-week washout period to minimize carryover effects. Each intervention phase lasted five consecutive days. The crossover design allowed each participant to serve as their own control, thereby improving power and reducing inter-subject variability.

The sample size was estimated for the primary within-participant change in TBUT. Olafsson et al. [22] reported a mean improvement of approximately 2.5 seconds following eyelid-warming treatment, while Sadri et al. [26] reported an observed standard deviation of 3.2 seconds for change in TBUT. Assuming an expected mean change of 2.5 seconds, an SD of 3.2 seconds, a two-sided paired-samples t-test, α = 0.05, and 80% power, the standardized effect size was 0.78 and the minimum required sample was 14 participants. Accordingly, 15 participants were recruited, all of whom completed both intervention periods. The ethics-approved protocol initially proposed recruitment of up to 50 participants; the final sample of 15 reflected recruitment and follow-up feasibility and is reported as protocol deviation in S3 Protocol.

### 2.3 Participants

Fifteen adults aged 18–40 years with a clinical diagnosis of DES associated with MGD were recruited from the university’s optometry clinic (S1 Fig.). Eligibility required an Ocular Surface Disease Index (OSDI) score ≥13 and a tear break-up time (TBUT) ≤10 seconds in at least one eye. Participants with active ocular infections, eyelid or conjunctival disease other than MGD, recent ocular surgery within six months, systemic conditions or medications known to affect tear film (e.g., beta-blockers, antihistamines), or habitual contact lens wear were excluded. Participants were instructed to discontinue any other dry eye therapies during the study.

### 2.4 Intervention protocol

Participants underwent two eyelid warming interventions using commercially available, clinically recognized devices. For the USB-Powered warm compress device, participants used the Wizard Research Heated Eye Mask (Wizard Research, United Kingdom; available at wizardresearch.co.uk). Per manufacturer report, it represents a new generation of smart eyelid-warming devices with features designed to enhance user convenience, thermal precision, and safety. Although, up to the best of our knowledge, peer-reviewed studies evaluating this device are not yet available, manufacturer-reported data indicate that approximately 93% of surveyed users experienced symptom relief, 77.6% improvement in quality of life, and 73% improvement in productivity, suggesting high consumer satisfaction. However, these claims have not been independently validated in controlled clinical trials. The device offers several smart features that distinguish it from conventional compresses. It includes adjustable temperature settings, allowing users to select precise therapeutic heat levels (typically 37°C to 47°C), thus maintaining eyelid temperatures sufficient for meibum liquefaction. A built-in timer permits users to set session durations in 10-minute intervals enhancing both safety and adherence. The device is USB-powered, offering portability through connections to laptops or power banks without the need for reheating, making it especially useful for users with active lifestyles. According to manufacturer its carbon fiber heating elements promote even heat distribution, reducing the risk of hot or cold spots, while the contoured, soft-fabric design conforms closely to the periocular region, improving thermal contact and user comfort. Together, these features aim to address the limitations of traditional compresses, including inconsistent heating, lack of temperature control, and patient non-compliance. The USB-Powered mask was applied bilaterally for 10 minutes once daily between 8 AM and 3 PM.

For the self-heating mask, participants used the disposable self-heating eye masks (dimensions: 18.5 × 8 cm; Hebei Xianrong Medical Equipment Co., Ltd., Hebei Province, China) consist of a heating element enclosed within a structural support. The heating component comprises a sheet containing iron powder, activated carbon, salt, and water-absorbing resin, covered by a breathable outer membrane (Fig 1). Heat is generated through an exothermic oxidation reaction of iron. According to manufacturer the reaction typically lasts around 20 minutes, maintaining an average surface temperature of approximately 41 °C. Per manufacturer, for optimal thermal effect, the mask must be removed from its sealed packaging immediately before application to initiate the heat-generating process. The self-heating mask was applied bilaterally for 10 minutes once daily between 8 AM and 3 PM.

**Fig 1:**
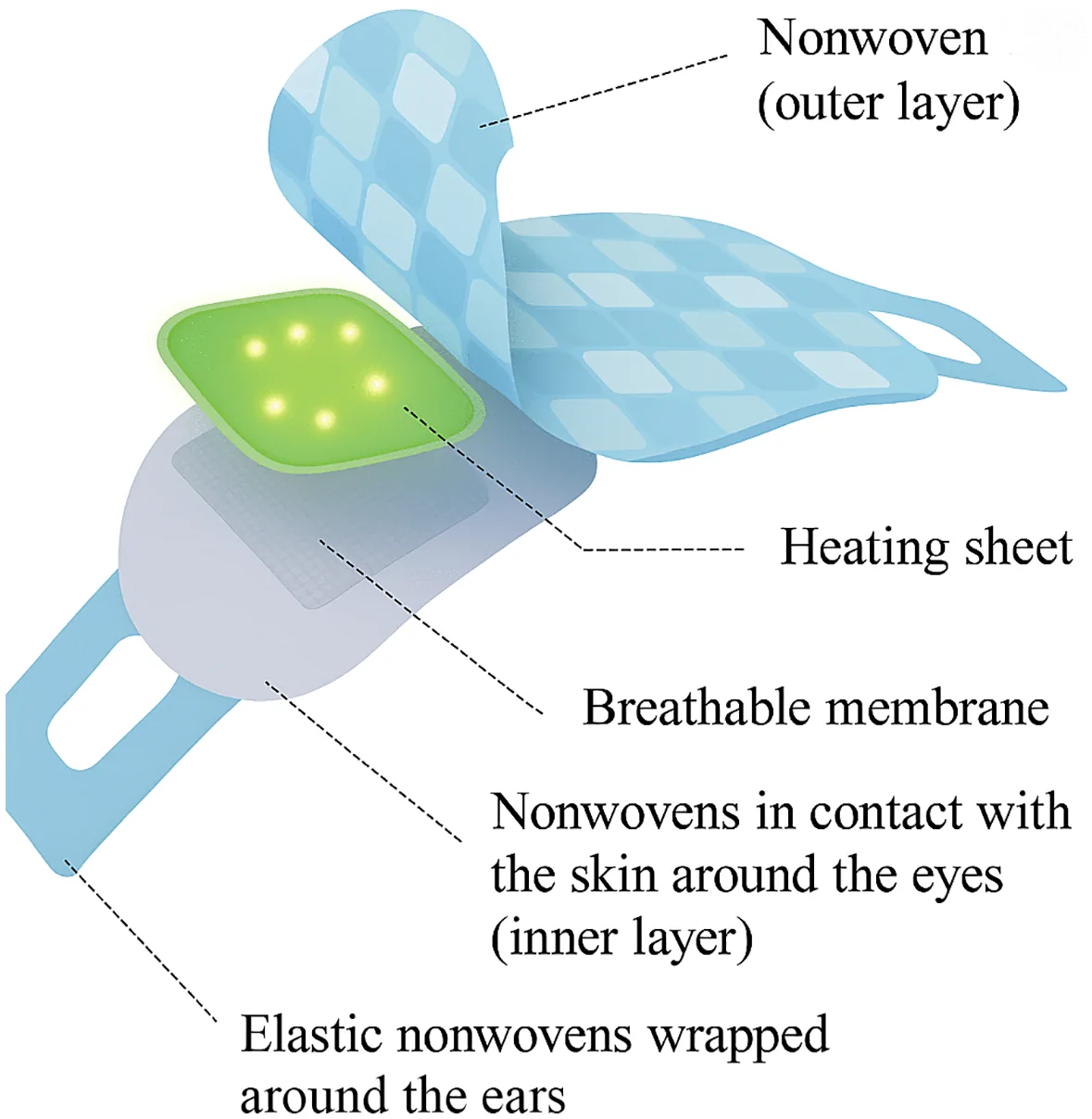
Structural design of the self-heating eye mask.

Both interventions were scheduled once daily for five consecutive days, with a two-weeks washout period before crossover to the alternate device. To document compliance, the treatment sessions took place at the optometry clinic, and participants rested with their eyes closed and avoided additional dry eye therapies throughout full study period. No deviations from the ethics committee–approved study protocol occurred during the conduct of the trial, and no adverse events were reported.

### 2.5 Outcome measures

The prespecified primary outcome was non-invasive TBUT. Additional objective outcomes included tear meniscus height (TMH) and visual acuity. TBUT and TMH were measured using the Oculus Keratograph 4M (Oculus Optikgeräte GmbH, Wetzlar, Germany), and visual acuity was measured using an ETDRS chart (Precision Vision Inc., La Salle, Illinois, USA). Patient-reported outcomes were also assessed with the OSDI and Standard Patient Evaluation of Eye Dryness (SPEED) questionnaires. All tests were performed in a controlled room environment.

### 2.6 Data analysis

Data were analysed using IBM SPSS Statistics (IBM Corp., Armonk, NY, USA). Normality testing using the Shapiro–Wilk test confirmed that TBUT, and TMH values were normally distributed (TBUT: W = 0.939, *p* = 0.107; TMH: W = 0.953, *p* = 0.140; SPEED: W = 0.945, *p* = 0.121). No significant outliers were detected for any variable. Accordingly, two-way repeated measures ANOVA examined time (Days 1–5) and device effect (USB-powered compress vs. self-heating mask), Post-hoc Bonferroni tests adjusted for multiple comparisons. Descriptive statistics are reported as mean ± standard deviation (SD). Statistical significance was set at p < 0.050.

## 3 Results

All 15 participants (mean age 22.27 ± 1.60 years) completed both intervention phases with full protocol adherence and no reported adverse events. Baseline characteristics were balanced, with a mean OSDI score of 19.2 ± 5.3, indicating mild to moderate dry eye, and mean baseline TBUT of 7.2 ± 1.5 s. A detailed summary of all outcome measures, including within-group and between-device comparisons, is presented in Table 1. Visual acuity remained stable across both phases, with no significant differences observed (0.00 ± 0.02 logMAR).

**Table 1:** Comparison of mean outcome measures for the USB-powered and Self-Heating warm Compresses.

Outcome Measure	Time Point	USB-Powered Compress (Mean ± SD)	Self-Heating Mask (Mean ± SD)	Within- Device (Baseline Vs Day 5)	Between-Devices (at Day 5)
TBUT (s)	Baseline	7.2 ± 1.5	7.3 ± 1.4	—	—
TBUT (s)	Day 3	8.8 ± 2.0	8.3 ± 1.9	USB-Powered: p = 0.014*; Self-Heating: p = 0.018*	—
TBUT (s)	Day 5	9.5 ± 2.2	7.9 ± 2.3	USB-Powered: p = 0.004*; Self-Heating: p = 0.082	p =0.026*
TMH (mm)	Baseline	0.16 ± 0.05	0.16 ± 0.05	—	—
TMH (mm)	Day 3	0.25 ± 0.03	0.21 ± 0.04		
TMH (mm)	Day 5	0.28 ± 0.07	0.22 ± 0.06	USB-Powered: p = 0.007*; Self-Heating: p = 0.039*	p =0.037*
OSDI	Baseline	19.2 ± 5.3		—	—
SPEED	Baseline	7.1 ± 1.8		—	—
SPEED	Day 5	5.6 ± 1.7	5.9 ± 1.4	USB-Powered: p = 0.71; Self-Heating: p = 0.80	p = 0. 59

TBUT, Tear break-up time; TMH, Tear meniscus height; OSDI, Ocular Surface Disease Index; SPEED, Standard Patient Evaluation of Eye Dryness; *indicate statistically significant

Both the USB-powered warm compress and the self-heating mask demonstrated improvement in tear film stability and tear volume over the five-day application period, although the degree and trend of improvement varied between devices. Across observed visits, the USB-powered device was associated with higher TBUT (likelihood-ratio χ^2^(1) = 7.43, p = 0.006) and TMH (χ^2^(1) = 4.49, p = 0.034). Further, repeated measures ANOVA revealed a significant main effect for time on TBUT (*F*(4,56) = 8.43, *p* < 0.001) and a significant time-by-device interaction (*F*(4,56) = 2.93, *p* = 0.028) (Table 1). Specifically, the mean TBUT in the USB-Powered compress phase increased steadily from 7.2 ± 1.5 seconds at baseline to 8.8 ± 2.0 seconds by Day 3, and further to 9.5 ± 2.2 seconds by Day 5 (Table 1 and Fig 2). In comparison, the self-heating mask phase showed a similar initial rise of TBUT to 8.3 ± 1.9 seconds at Day 3 but then plateaued, with the Day 5 mean decreasing slightly to 7.9 ± 2.3 seconds (Table 1 and Fig 2). A within-device comparison confirmed that the TBUT increase from Day 3 to Day 5 was statistically significant for the USB compress (*t*(14) = 2.48, *p* = 0.026), while no significan*t* additional change was observed for the self-heating mask (*t*(14) = 1.12, *p* = 0.280) (Table 1).

**Fig 2:**
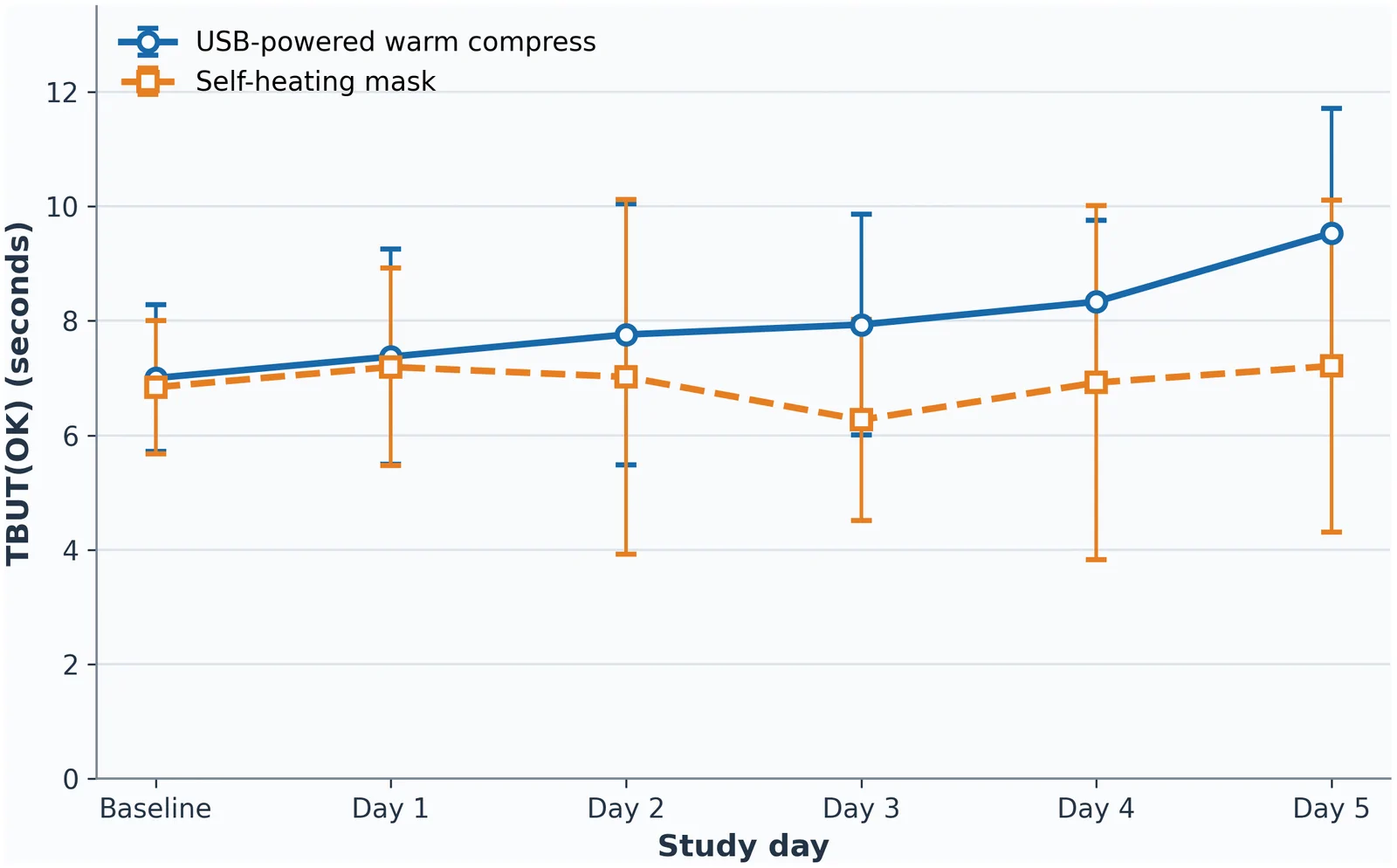
Daily trend in mean TBUT for the USB-powered warm compress and the self-heating eye mask from baseline to Day 5 time point. The USB-powered compress produced a steady increase in tear film stability across all five days, whereas the self-heating mask showed an early improvement that plateaued after Day 3.

Tear Meniscus Height (TMH) increased in both intervention phases, with the USB compress showing a greater magnitude of change overall (Table 1). Mean TMH rose from 0.16 ± 0.05 mm at baseline to 0.28 ± 0.07 mm by Day 5 for the USB-powered warm compress, compared to 0.22 ± 0.06 mm for the self-heating mask. Repeated measures ANOVA indicated a significant effect of device on TMH outcomes (*F*(1,14) = 5.12, *p* = 0.037) (Fig 3). The day-to-day trend for TMH in the USB phase showed a steady upward progression across all five days, whereas the self-heating mask effect appeared to plateau after Day 3 (Fig 4).

**Fig 3:**
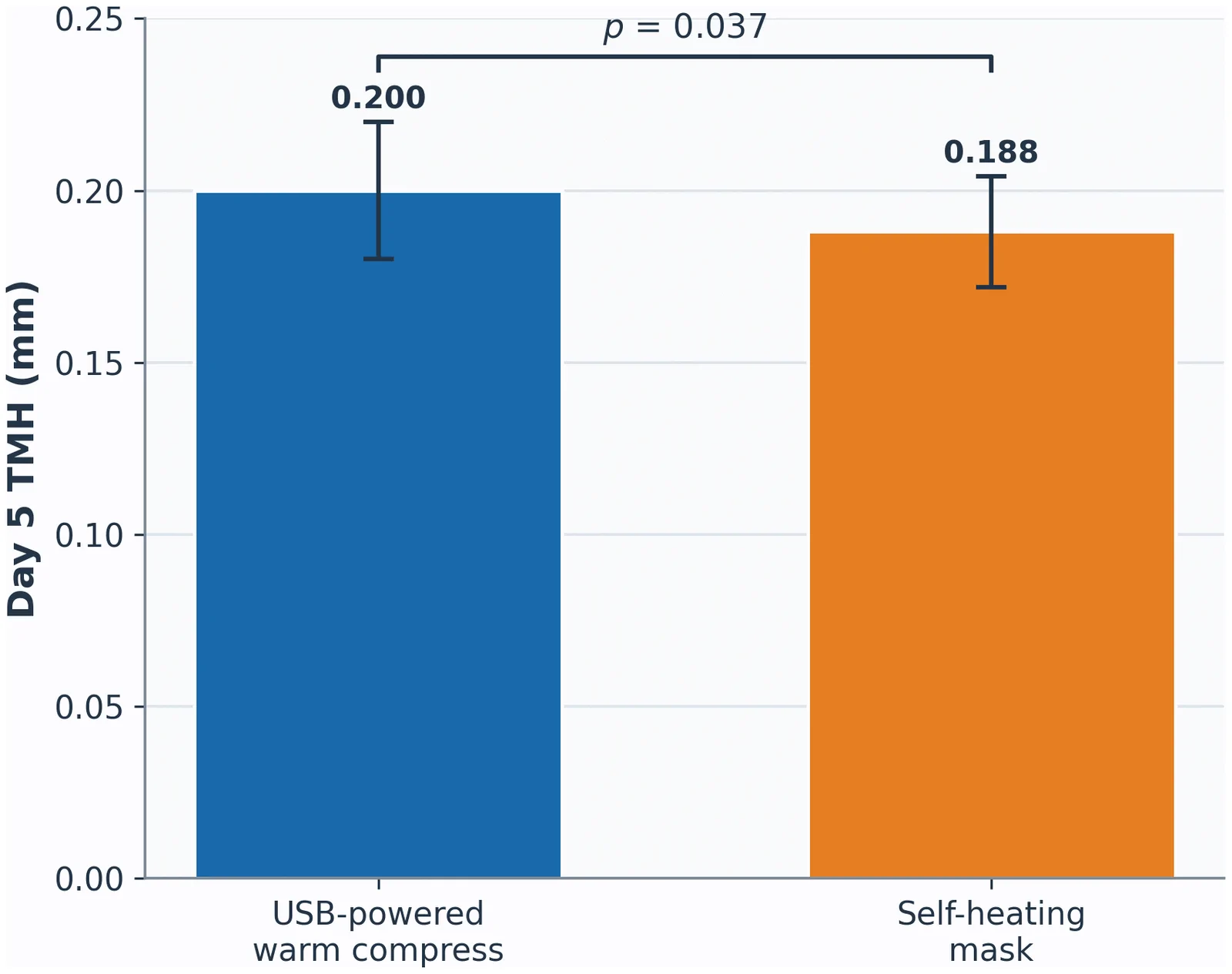
Descriptive Day 5 TMH for the USB-powered warm compress and self-heating mask. Bars show means and error bars show standard deviations.

**Fig 4:**
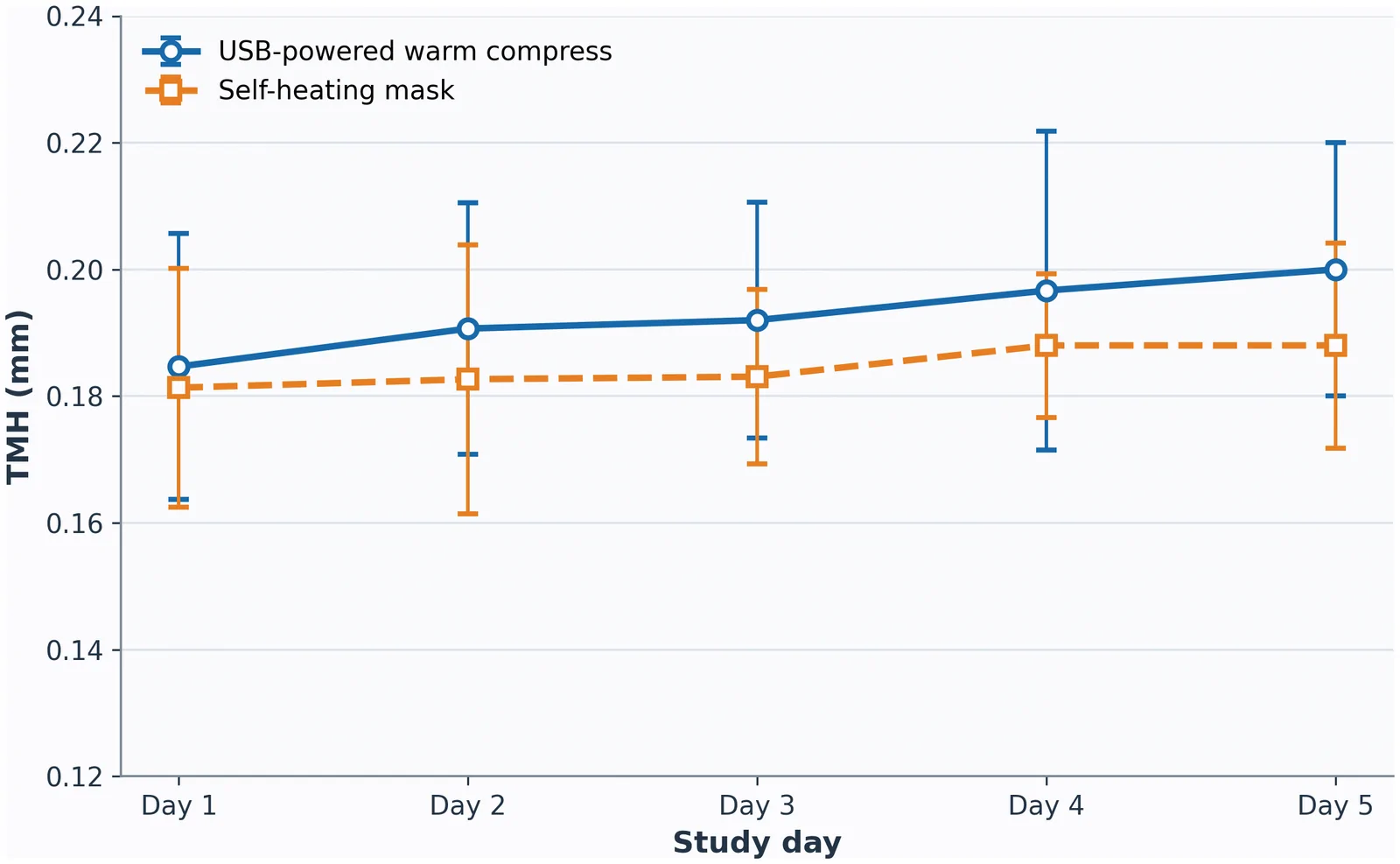
Daily trend in mean TMH from Day 1 through Day 5 for the USB-powered warm compress and self-heating mask. Error bars indicate standard deviation. The USB-powered compress showed a steady increase in tear volume, while the self-heating mask plateaued after Day 3.

Both devices produced some improvements in patient-reported scores. The SPEED scores decreased from baseline in both phases, with a mean reduction from baseline in both phases (USB-powered warm compress: –1.5 ± 0.80; self-heating mask: –1.2 ± 0.70) but no significant difference between devices was observed (*t*(14) = 0.95, *p* = 0.355). Participants consistently reported higher satisfaction with both interventions, although qualitative feedback indicated a preference for the USB-powered warm compress due to its consistent warmth and ease of reactivation despite similar SPEED scores.

When comparing the effect of three days versus five days of use, the USB-powered warm compress demonstrated a continuous improvement in TBUT and TMH between Day 3 and Day 5, confirming a continued therapeutic effect beyond the third day. In contrast, the self-heating mask phase showed no significant additional improvement after Day 3, suggesting a plateau in its therapeutic impact. However, these differences were not statistically significant.

Overall, these findings indicate that both eyelid warming modalities were effective in improving tear film stability, tear volume, and subjective comfort. However, the USB-powered warm compress provided greater and more sustained improvements in TBUT and TMH across the full five-day period compared to the self-heating mask.

## 4 Discussion

This randomized crossover clinical trial demonstrates that both the USB-powered warm compress and the self-heating mask are effective short-term interventions for managing evaporative dry eye disease (DED) associated with meibomian gland dysfunction (MGD). However, the USB-powered device consistently produced greater improvements in objective tear film stability and tear volume across five days of use, reinforcing the clinical principle that sustained, adjustable eyelid warming is more effective for liquefying meibum and restoring tear film integrity. These findings are consistent with the TFOS DEWS II reports, which emphasize that maintaining eyelid temperatures of at least 40°C for sufficient durations is necessary to melt viscous meibum and support lipid layer quality [12,19,20].

In our study, TMH increased substantially in the USB-powered warm compress phase but less so with the self-heating mask, which supports the idea that more stable lipid layers reduce evaporation and help maintain tear reservoir volume [13,14]. This trend agrees with the work of Finis et al. (2014) [26] and Suzuki et al. (2022) [14], who noted that consistent heat can improve gland expressibility and aqueous retention over time.

Both interventions improved patient-reported symptoms (SPEED), but the absence of a significant difference between devices suggests that perceived symptom relief may be comparable, despite measurable differences in objective tear film parameters. This highlights an important aspect of warm compress therapy: practical comfort, convenience, and user preference strongly influence adherence, which is crucial for sustained benefit. Our participants subjectively reported a better preference for the USB-powered compress due to its consistent warmth and ease of reactivation, supporting observations from patient surveys that convenience and thermal performance are key to daily compliance [24,25]. Recent consumer health reports and clinical reviews further confirm that when heat delivery is perceived as ineffective or inconvenient, patients are less likely to maintain the recommended daily routine [24,25]. Patient-centered barriers such as lack of time, forgetfulness, and misunderstanding of treatment importance significantly impacted compliance with daily warm compress therapy [25]. Studies have shown that while only ~55% of patients adhere to twice-daily lid hygiene [25], the use of device-based warm compresses (e.g., EyeGiene®, Blephasteam®) may help improves adherence compared to traditional towels, although direct adherence data remains limited [27]. Although we did not observe a statistically significant difference in SPEED scores between devices, the trend favouring the USB compress is promising and may become significant with longer treatment durations, as reported in studies with extended follow-up [19,20,22].

Our results contrast with recent studies [21,22] that reported comparable clinical outcomes between various warm compress modalities, including both electrically powered and non-electrical devices. Wang et al. (2015) [21] found that microwavable masks provided more consistent heating than disposable chemical masks, although with comparable outcomes. Olafsson et al. (2021) [22] showed that both an electrically powered steam mask and a non-electric gel mask significantly improved TBUT and meibum expressibility, with no clear superiority between them. Notably, Blackie et al. (2008) demonstrated that warm compress effectiveness varies by heating method and correlates with lid surface temperatures [28], supported by Blackie & Korb’s (2009) finding that temperatures ≥40°C for ≥5 minutes are critical for meibum liquefaction [29]. Similarly, Magno et al. (2022) [20] demonstrated that while single-use steam masks are convenient, they tend to cool rapidly under ambient conditions, particularly in air-conditioned settings common in the Middle East. This practical limitation likely explains the plateau effect we observed with the self-heating mask: its benefit peaked by Day 3, whereas the USB-powered compress continued to improve TBUT and TMH through Day 5. Such findings are clinically relevant in regions where fluctuating indoor temperatures and low humidity exacerbate tear evaporation [4,5,7]. The discrepancy between our findings and previous studies [21,22] may be attributed to several methodological and device-related differences. Unlike Blephasteam, TheraPearl, or similar modalities the Wizard USB-powered device used in our study offers adjustable, sustained heating with consistent thermal delivery, which may enhance meibum softening and improve gland function. In contrast, self-heating masks rely on passive chemical reactions that may not maintain therapeutic temperatures long enough to achieve similar effects. Additionally, Olafsson et al. (2021) found no significant differences between Blephasteam and TheraPearl in improving TBUT or gland expressibility; however, both groups in their study concurrently used artificial tears, which may have diluted the comparative effect of the warm compress therapies. Furthermore, differences in population severity, adherence, and protocol design could have influenced outcomes. On the other hand, Wang et al. (2015) evaluated only the thermal characteristics of microwavable and chemical-heat masks in healthy individuals, without assessing any clinical parameters such as tear stability or volume. The absence of adjunctive treatments in their study allowed for isolated evaluation of heat retention but limits direct comparison with patient-centered therapeutic outcomes. Taken together, these distinctions in device design, patient populations, adjunctive therapy, and outcome measures may explain why our findings contrast with earlier reports.

From a clinical perspective, this study findings emphasize that patients with mild to moderate MGD, especially in arid climates with variable indoor cooling, may benefit more from warm compresses that delivers stable, adjustable heat compared to single-use self-heating masks [17,19–21]. Although microwave and self-heating masks are convenient and affordable, their tendency to cool quickly can limit treatment efficacy if not reheated properly [19,20]. For patients with busy lifestyles or prolonged screen exposure in dry indoor environments, a USB-powered device with precise temperature control could provide better results with fewer interruptions [5,19,24,25]. Clinicians should include detailed usage instructions and demonstrate proper application during consultations to enhance adherence, as many patients struggle to maintain adequate eyelid contact or heat duration on their own [24,25]. This newer USB-powered device (Wizard Research Heated Eye Mask) or similar future devices have some smart features including adjustable temperature settings and built-in heating control to help maintain consistent eyelid temperatures, potentially supporting compliance [24,25].

Equally important are cost considerations and long-term value. While electrical devices often require higher upfront investment than disposable masks, their reusability and reliable performance may reduce the need for multiple daily treatments, additional lubricants, and repeated clinic visits — ultimately offsetting costs for patients in high-prevalence regions like Saudi Arabia and the Gulf states [11,30]. Eye care professionals should guide patients through this cost–benefit tradeoff as part of shared decision-making. The electrically powered devices cost ~ $100 vs. $5 per single-use self-heated mask, the reusability of electrically powered devices may reduce long-term expenses for patients. Though direct cost comparisons between electrically powered and microwave masks are unavailable, two principles emerge: (1) ineffective therapy increases long-term expenditures [31], and (2) reusable devices could improve adherence, reducing indirect costs [1,24,25]. Our finding that electrically powered devices showed superior efficacy suggests potential economic advantages worthy of future formal analysis. Warm compress therapy should also be prescribed within a broader MGD management plan that includes lid hygiene, dietary modification, or prescription agents when appropriate [1,2,29,30]. Matching device features to individual patient needs, preferences, and living environments is vital for maximizing real-world impact.

This study has several limitations that should be acknowledged. First, the five-day intervention duration limits insight into long-term gland recovery and adherence trends, which typically require several weeks to assess [17,20]. Second, although the priori paired-outcome calculation required 14 participants, the sample remained small and did not support subgroup analyses. Third, we relied on self-reported compliance and did not directly measure eyelid surface temperatures during use, which could lead to underestimation of heat loss in certain conditions especially with the self-heating masks [21]. Lastly, additional diagnostic tools such as meibography, tear osmolarity testing, or inflammatory biomarker analysis were not included, which would provide a more comprehensive view of ocular surface changes [14].

Future research should extend follow-up durations to at least four weeks to assess whether the superior performance of USB-powered compresses is sustained over time. Trials integrating non-contact meibography [32] or tear osmolarity testing [33] could elucidate structural and biochemical changes underlying TMH improvements. Large, multicenter studies could enable robust subgroup analyses and cost-effectiveness evaluations, helping clinicians understand which patient groups benefit most from different warming modalities. Comparative trials could also explore next-generation devices, which may address practical barriers like inconsistent heating in variable ambient environments. Additionally, future studies could also examine patient education strategies, behavioral interventions, and personalized device recommendations to optimize long-term adherence and reduce drop-off rates. Addressing these research gaps will be essential for reducing the personal and economic burden of evaporative DES, which remains a growing public health challenge in the Middle East and worldwide [4,5,10].

In summary, this randomized crossover study demonstrates that a USB-powered electrical warm compress delivers more consistent short-term improvements in tear film stability and tear volume compared to a self-heating mask, without compromising subjective symptom relief or safety. These results highlight the clinical value of sustained thermal performance, ease of use, and proper patient education in optimizing home-based therapies for MGD. As the prevalence of dry eye disease continues to rise globally and within the Middle East, incorporating reliable warming technologies into daily care regimens could enhance treatment outcomes and improve quality of life for patients with chronic evaporative dry eye.

## 5 Conclusions

This randomized crossover study shows that a USB-powered warm compress provides more consistent improvements in tear film stability and tear volume than a self-heating mask in patients with evaporative dry eye disease linked to meibomian gland dysfunction. Both devices improved symptoms, but the USB-powered device maintained therapeutic heat more effectively and showed sustained benefit over five days. These results highlight the value of thermal performance and user convenience in eyelid warming therapies. Further research is needed to assess long-term outcomes, gland recovery, and strategies to improve adherence and cost-effectiveness.

## Supporting information

S1 Fig CONSORT flow diagram of participant enrolment, randomization, crossover allocation, follow-up, and analysis. (TIF)

S2 Checklist CONSORT 2025 checklist. (DOCX)

S3 Protocol Institutional Authorization to Publish Trial Protocol. (PDF)

S4 File Trial protocol for publication under CC BY 4.0. (DOCX)
