# Supplementary material for: USB-powered vs. self-heating warm compresses for meibomian gland dysfunction: A prospective randomized crossover clinical trial
Source: PLoS One. 2026 Oct 13;21(10):e0360762. doi: 10.1371/journal.pone.0360762 (PMC13660908; doi:10.1371/journal.pone.0360762)
Supplement: Supplementary file 1 [file pone.0360762.s003.pdf]

Institutional Authorization to Publish Trial Protocol

Signature-ready statement for PLOS ONE

Manuscript ID: PONE-D-26-21759
IRB approval: E-25-9589

To: The Editors of PLOS ONE

King Saud University authorizes the clean protocol for the study entitled “USB-Powered vs. Self-Heating Warm Compresses for Meibomian Gland Dysfunction: A Prospective Randomized Crossover Clinical Trial” to be published as Supporting Information with the associated PLOS ONE article, if accepted.

The institution understands that the protocol will be published under the Creative Commons Attribution 4.0 International (CC BY 4.0) license and will be freely available for access, download, copying, distribution, adaptation, and reuse, including commercial reuse, provided appropriate attribution is given.

The version authorized for publication is the clean protocol labeled S3 Protocol. Confidentiality notices, institutional logos, and signatures have been removed from that publication copy. This authorization does not permit disclosure of identifiable participant information or other records outside the clean protocol.

Authorized institutional representative:

Name: __Prof. Ali Mazyed Alsaqr_______________

Title/Office: _Vice Dean for postgraduate studies and research, college of applied medical sciences

Institution: __King Saud University__________________

Signature: ____

Date: ____12/8/2026___________________
