# Supplementary material for: USB-powered vs. self-heating warm compresses for meibomian gland dysfunction: A prospective randomized crossover clinical trial
Source: PLoS One. 2026 Oct 13;21(10):e0360762. doi: 10.1371/journal.pone.0360762 (PMC13660908; doi:10.1371/journal.pone.0360762)
Supplement: Supplementary file 3 [file pone.0360762.s004.docx]

# S2 Trial Protocol

**Complete and detailed ethics-approved clinical trial protocol from the original King Saud University IRB-approved study documents (E-25-9589).**

**Protocol Confirmation**

We confirm that this document represents the complete and detailed clinical trial protocol submitted to and approved by the King Saud University Institutional Review Board (KSU-IRB) prior to the commencement of participant recruitment, enrollment, and study procedures. The protocol was originally prepared in English as part of the ethics submission package and reflects the approved scientific rationale, study objectives, intervention procedures, outcome measures, safety considerations, and statistical analysis plan underlying the conducted clinical trial.

## Study Title

USB-Powered vs. Self-Heating Warm Compresses for Meibomian Gland Dysfunction: A Prospective Randomized Crossover Clinical Trial

## Principal Investigator

Prof. Ali Alsaqr
Department of Optometry
College of Applied Medical Sciences
King Saud University
Riyadh, Saudi Arabia

## Background and Scientific Rationale

Dry eye disease associated with meibomian gland dysfunction (MGD) is a multifactorial ocular surface disorder characterized by tear film instability, ocular discomfort, fluctuating vision, and chronic ocular surface irritation. MGD contributes significantly to evaporative dry eye disease through abnormalities in meibomian gland secretion, resulting in lipid layer insufficiency and increased tear evaporation. Warm compress therapy is widely considered a first-line conservative treatment for MGD because therapeutic heat application improves meibum liquefaction, enhances glandular secretion, and improves tear film stability. Traditional warm compress methods, however, frequently suffer from inconsistent heat retention, poor thermal control, and limited patient adherence. USB-powered warm compresses and disposable self-heating eye masks have recently been introduced to improve temperature consistency and treatment convenience. Despite increasing clinical use of these devices, direct comparative evidence between these modalities remains limited. Therefore, this randomized crossover clinical trial was designed to compare the effectiveness of a USB-powered warm compress and a self-heating warm compress in improving objective tear film parameters and subjective dry eye symptoms in patients with MGD-associated evaporative dry eye disease.

## Study Objectives

The primary objective of this study was to compare the effectiveness of USB-powered warm compresses and self-heating warm compresses in improving tear film stability in patients with meibomian gland dysfunction-associated dry eye disease. Secondary objectives included evaluating changes in tear break-up time (TBUT), tear meniscus height (TMH), patient-reported dry eye symptoms measured using the Ocular Surface Disease Index (OSDI) and Standard Patient Evaluation of Eye Dryness (SPEED) questionnaires. The study also aimed to evaluate participant adherence and subjective treatment satisfaction associated with each intervention modality.

## Study Design

This study was designed as a prospective randomized crossover clinical trial conducted at the Department of Optometry, College of Applied Medical Sciences, King Saud University, Riyadh, Saudi Arabia. Participants will be randomly assigned to one of two intervention sequences. In Group A, participants received the USB-powered warm compress first followed by the self-heating warm compress after completion of the washout period. In Group B, participants received the self-heating warm compress first followed by the USB-powered warm compress. The crossover design allowed each participant to serve as their own control, thereby minimizing inter-subject variability and improving statistical efficiency.

## Study Duration and Timeline

The study duration was planned for up to one year and included protocol preparation, IRB approval, participant recruitment and screening, intervention implementation, washout periods, data collection, statistical analysis, manuscript preparation, and dissemination of findings. Participant recruitment and follow-up procedures will be conducted between July 2025 and December 2025.

## Study Setting

All study procedures, ocular examinations, and intervention sessions will be conducted at the Optometry Clinic, Department of Optometry, College of Applied Medical Sciences, King Saud University, Riyadh, Saudi Arabia.

## Participant Recruitment and Eligibility

Participants will be recruited through advertisements distributed within the university optometry clinic and affiliated community settings. Adults aged between 18 and 40 years with clinically diagnosed evaporative dry eye disease associated with meibomian gland dysfunction will be screened for eligibility. Screening procedures included medical and ocular history review, tear film evaluation, and administration of the Ocular Surface Disease Index questionnaire. Inclusion criteria included an OSDI score greater than 13, tear break-up time less than 10 seconds, and ability to provide written informed consent. Exclusion criteria included active ocular infection, ocular surgery within the previous six months, contact lens wear during study participation, systemic diseases affecting tear production, medications known to influence tear film stability, and known allergy or intolerance to warm compress materials.

## Randomization and Allocation

Participants will be randomized into one of the two crossover intervention groups using a random allocation approach. Due to the physical characteristics of the interventions, masking was not feasible and the study was conducted as an open-label clinical trial. The crossover design ensured that all participants received both interventions during the study period.

## Intervention Procedures

Participants attended baseline and follow-up visits during each intervention phase. Baseline assessments included visual acuity evaluation, tear break-up time measurement, tear meniscus height assessment, and symptom assessment using the OSDI and SPEED questionnaires. Participants then received the allocated warm compress intervention for approximately 10 minutes. Objective ocular measurements will be repeated after treatment according to the study schedule. Each intervention phase consisted of repeated treatment sessions over five consecutive days. A two-week washout period separated the intervention phases to minimize carryover effects and allow ocular surface parameters to return toward baseline before crossover to the alternate intervention.

## Intervention Devices

The USB-powered warm compress consisted of a reusable USB-powered heated eye mask designed to maintain controlled therapeutic heat during treatment sessions (Wizard Research Heated Eye Mask, Wizard Research, United Kingdom; available at wizardresearch.co.uk). The self-heating warm compress consisted of a disposable self-heating mask that generated heat through a thermal activation mechanism after exposure to air (dimensions: 18.5 × 8 cm; Hebei Xianrong Medical Equipmwnt Co., Ltd., Hebei Province, China). Both interventions will be used according to manufacturer instructions and will be applied for approximately 10 minutes during each treatment session over five consecutive days.

## Outcome Measures

The primary outcome measure was tear break-up time (TBUT), which was used as an indicator of tear film stability. Secondary outcome measures included tear meniscus height (TMH), Ocular Surface Disease Index (OSDI) scores, and Standard Patient Evaluation of Eye Dryness (SPEED) scores. Outcome measurements will be collected before and after every intervention sessions according to the predefined study schedule.

## Instrumentation and Clinical Assessments

Clinical assessments will be conducted using validated ophthalmic instruments and standardized examination procedures. Best corrected visual acuity will be assessed using an ETDRS chart. Tear film assessments will be performed using Oculus Keratograph 5M® systems. Subjective dry eye symptoms will be evaluated using the Ocular Surface Disease Index (OSDI) questionnaire and the Standard Patient Evaluation of Eye Dryness (SPEED) questionnaire.

## Safety and Risk Management

Potential risks associated with study participation will be considered minimal. Participants could experience temporary periocular redness, mild skin irritation, transient blurred vision, or mild ocular discomfort following warm compress application. To minimize risks, all devices will be checked to ensure safe operating temperatures before use. Participants received detailed instructions regarding safe application procedures and will be advised to immediately report any discomfort or adverse effects. Study personnel will be available during all study visits to monitor participant safety. Participants retained the right to withdraw from the study at any time without penalty.

## Data Collection and Confidentiality

Clinical measurements and questionnaire responses will be recorded on standardized study forms and subsequently entered into a Excell database and then in SPSS for statistical analysis. Participant confidentiality will be maintained throughout the study using coded identifiers rather than personally identifiable information. Access to identifiable participant data will be restricted to authorized members of the research team only. All study procedures adhered to institutional ethical guidelines governing research involving human participants.

## Sample Size and Statistical Analysis

The original ethics-approved protocol proposes recruitment of up to 50 participants. During study implementation, and due to recruitment feasibility and participant adherence considerations, and the final completed study included 15 participants. Statistical analysis will be performed using SPSS statistical software. Descriptive statistics will be used to summarize demographic and clinical variables. Comparative analyses between interventions will be conducted using statistical tests appropriate for crossover trial designs. Statistical significance will be defined as p < 0.05.

## Ethical Considerations

The study protocol will be reviewed and approved by the King Saud University Institutional Review Board (KSU-IRB) before participant recruitment and data collection commenced. Written informed consent will be obtained from all participants before enrollment. The study adhered to the principles of the Declaration of Helsinki and institutional ethical standards governing research involving human participants. Participants will be informed about study procedures, potential risks and benefits, confidentiality protections, voluntary participation rights, and their right to withdraw from participation at any time without penalty.

## Protocol Deviations

The original ethics-approved protocol proposed recruitment of up to 50 participants and longer intervention duration. During study implementation, operational modifications will be introduced to improve recruitment feasibility and participant adherence. The final completed study included 15 participants and shorter intervention periods. These modifications did not alter the primary scientific objectives, intervention comparisons, outcome measures, or statistical analysis framework of the clinical trial. No major safety-related deviations occurred during the study.

## Trial Registration

ClinicalTrials.gov Identifier: NCT07618169

## Publication Statement

The authors confirm that this document corresponds to the ethics-approved clinical trial protocol submitted before study commencement. The authors agree that this protocol may be made available to editors and reviewers and published as Supporting Information if the manuscript is accepted for publication.
